# Predictors of rehospitalization due to violent behavior in patients with psychotic disorders with a history of violent behavior

**DOI:** 10.3389/fpsyt.2025.1624706

**Published:** 2025-08-01

**Authors:** Ulaş Korkmaz, Meltem Hazel Şimşek

**Affiliations:** Department of Psychiatry, Giresun University, Giresun, Türkiye

**Keywords:** psychotic disorders, violent behavior, treatment adherence, forensic psychiatry, rehospitalization

## Abstract

**Background:**

Some individuals with psychotic disorders may exhibit violent behavior, necessitating psychiatric hospitalization to ensure both patient care and public safety. Understanding factors behind post-discharge rehospitalization due to violence is crucial. This study aims to examine the association between treatment adherence, psychiatric follow-up frequency, and prescribed medications with the risk of violent-behavior-related rehospitalization in patients with psychotic disorders.

**Method:**

This retrospective cohort study included 68 patients diagnosed with psychotic disorders under mandatory forensic psychiatric follow-up between January 2022 and February 2025. Patients were categorized into two treatment groups: oral antipsychotic treatment and long-acting injectable (LAI) antipsychotic treatment. The primary outcome was rehospitalization due to violent behavior. Binary logistic regression and Cox regression analyses were performed to identify predictors of rehospitalization and treatment adherence.

**Results:**

The mean age was 46.76 years. 80.9% of the patients were male, and 70.6% were adherent to treatment. During the follow-up period, 14.7% of the patients were rehospitalized due to violent behavior. Non-adherent patients had a significantly higher risk of rehospitalization (p < 0.001), with a 15-fold increased risk compared to adherent patients. While LAI antipsychotic use, regular follow-up at Community Mental Health Centers, and more frequent psychiatric evaluations were associated with lower rehospitalization rates, these effects did not reach statistical significance.

**Conclusions:**

Treatment adherence is a key factor in preventing rehospitalization due to violent behavior. Given its substantial predictive value, interventions targeting adherence, such as LAI antipsychotic use and psychosocial support, should be prioritized. Future studies should include objective medication adherence measures, long-term follow-up, and additional clinical outcomes.

## 1 Introduction

Psychotic disorders are chronic mental disorders that can lead to severe functional impairment and disability. However, not all patients with psychotic disorders follow the same clinical course. In some patients, violent behavior may be more pronounced. Compared to the general population, patients with psychotic disorders have a slightly increased risk of exhibiting violent behavior; however, this risk has been reported to be more pronounced in specific subgroups (1). Studies have shown that being in the active phase of the disorder, having poor treatment adherence, lacking insight, and having a history of substance use are significant factors that increase the likelihood of violent behavior in patients (2). In patients with psychotic disorders who exhibit recurrent violent behavior, the likelihood of engaging in violent acts and the subsequent need for involuntary psychiatric hospitalization increase when regular psychiatric follow-up and adequate psychiatric treatment are not ensured (3). This situation not only negatively impacts the clinical course of patients but also has serious implications for public health.

Involuntary hospitalization is typically implemented when an individual poses a serious risk to themselves, their family, or their surroundings. Many countries have legal regulations that mandate treatment and hospitalization for individuals with severe mental disorders who pose a risk to others. In Turkey, this process is regulated under the Turkish Civil Code. The civil code allows for the involuntary hospitalization of individuals diagnosed with psychotic disorders who pose a risk to society (4, 5). The Turkish Penal Code mandates that individuals with mental disorders who commit crimes receive treatment in forensic healthcare institutions and remain under supervision as long as they pose a risk to society. Following discharge, once patients have recovered and their risk to society has diminished, it is essential that they remain under follow-up monitoring and continue their treatment. Under the Turkish Penal Code, patients who have been discharged after receiving hospital treatment are subject to mandatory forensic psychiatric follow-up at regular intervals. If necessary, the individual is brought in for evaluation under the escort of law enforcement officers. Suppose patients exhibit violent behavior again due to their mental disorder, meaning they are deemed a renewed threat to society. In that case, the execution judge may order their readmission to the hospital based on medical board reports (6). Many countries have laws regulating mandatory treatment for individuals with mental disorders who pose a risk of violence and aggression. These laws allow involuntary treatment under specific conditions to ensure public safety and patients’ well-being (7). This legal process ensures that individuals with a history of aggression or violence due to mental disorders are monitored within the healthcare system and, if necessary, rehospitalized for treatment. Thus, potential risks for both patients and society are minimized. However, the roles of treatment adherence, regular psychiatric follow-up, and social support in this process remain unclear.

In patients with psychotic disorders, treatment adherence is one of the most critical factors influencing both the course of the disorders and the risk of violent behavior. Antipsychotic treatment is crucial for controlling violent behavior and preventing relapse of the disorder. In patients with irregular medication use, symptoms may worsen, the disorder may relapse, and the risk of violent behavior may increase. However, a significant portion of patients do not adhere to their prescribed medication regimen after discharge. Specifically, within the first year after discharge, approximately 50% of patients with schizophrenia have been found to either fail to adhere to their treatment regimen or discontinue it entirely (3, 8). A cohort study found that 15.7% of patients who discontinued antipsychotic treatment exhibited at least one instance of violent behavior during the follow-up period. In contrast, this rate was 8.3% among those who adhered to treatment regularly (9).

Some antipsychotic medications are more effective than others in controlling aggression and violent behavior. Clozapine is considered one of the most effective options for patients who do not respond adequately to other treatments or exhibit severe aggression. Clozapine stands out not only for its antipsychotic effects but also for its significant reduction in the risk of aggression. Numerous studies have demonstrated that clozapine significantly reduces aggression and hostility symptoms (1). However, due to the need for close monitoring and potential side effects associated with clozapine treatment, long-acting injectable (LAI) antipsychotics have become an important alternative for some patients (10). Research indicates that medication non-adherence is common among patients using oral antipsychotics, which, in turn, increases hospitalization rates. Therefore, LAI antipsychotics have become a crucial alternative for patients who use their medication irregularly or refuse oral treatment. Studies have shown that LAI antipsychotics not only reduce the risk of relapse and rehospitalization but also decrease levels of violence and aggression (10, 11). Follow-up studies on patients who initiated long-acting paliperidone palmitate treatment reported a 26% reduction in arrest rates compared to the previous year (3). These findings suggest that LAI antipsychotics can improve treatment adherence, thereby reducing violent behavior.

One of the effective factors in enhancing treatment adherence and reducing the risk of violence in patients with psychotic disorders is psychosocial support and community-based mental health services. In Turkey, Community Mental Health Centers (CMHCs) provide psychosocial support, rehabilitation, and regular follow-up for individuals with severe mental disorders, ensuring the continuity of treatment. CMHCs and similar centers facilitate regular follow-up and treatment continuity, thereby improving the course of the disorder and reducing the risk of aggression (12). Globally, community-based approaches have emerged as an effective strategy in preventing reoffending and aggressive incidents among individuals with severe mental disorders (13).

Although numerous studies have examined the factors associated with violent behavior in individuals with psychotic disorders, predictors of violence-related rehospitalization among patients under forensic psychiatric follow-up remain insufficiently explored. Therefore, this study aims to investigate the relationship between post-discharge treatment adherence, regular psychiatric follow-up, and prescribed treatments with the risk of rehospitalization due to recurrent violent behavior in individuals who were involuntarily hospitalized for violent behavior. This three-year retrospective cohort study examines post-discharge treatment processes, prescribed medications, and the continuity of regular psychiatric follow-up in patients.

The findings of this study are expected to contribute to the identification of high-risk psychotic disorder patients for aggression, the selection of appropriate treatment approaches, and the improvement of long-term patient follow-up.

## 2 Methodology

### 2.1 Study design and data collection

The present study is designed as a retrospective cohort study. The study sample consists of individuals diagnosed with psychotic disorders who were under mandatory forensic psychiatric follow-up under the Turkish Penal Code at a tertiary hospital’s psychiatric outpatient clinic between January 2022 and February 2025. Individuals diagnosed with schizophrenia, schizoaffective disorder, delusional disorder, brief psychotic disorder, schizophreniform disorder, or other specified/unspecified psychotic disorders were included in the study. In contrast, those diagnosed with bipolar disorder, dementia, or intellectual disability were excluded. After obtaining the necessary institutional approvals, patient data were collected from medical records, the hospital information system, and national electronic health records. These data include the patients’ ages at the initiation of forensic psychiatric monitoring, gender, marital status, post-discharge and current treatments, treatment adherence, regular utilization of CMHC services, follow-up frequency, and duration of follow-up.

Patients were divided into two groups based on their treatment regimen: (1) Oral treatment group, patients receiving only oral antipsychotic treatment, and (2) LAI group, patients receiving both oral and long-acting injectable (LAI) antipsychotic treatment. The Proportion of Days Covered (PDC) method assessed patients’ treatment adherence. PDC is a parameter calculated by dividing the number of days the medication was obtained by the total analyzed period, providing an estimate of the extent to which prescribed medications were used over a specific timeframe. PDC provides a quantitative measure of medication adherence (14). This study calculated PDC values based on the medications prescribed to patients after discharge. A PDC ≥ 0.8 was considered adherent to treatment, while a PDC < 0.8 was classified as non-adherent. Patients who attended CMHC follow-ups every 1 to 3 months were considered regularly utilizing CMHC services. In the overall sample, the recommended mandatory forensic psychiatric follow-up frequency after discharge was categorized into two groups: every three or six months. Follow-up durations were determined by the months between the patients’ first mandatory evaluation after discharge and the study endpoint. Data from discharge to the study endpoint were analyzed for each patient. Two different study endpoints were defined: (1) Rehospitalization due to violent behavior or (2) No recurrence of violent behavior until February 2025/Discontinuation of mandatory examinations at any time due to improvement in symptoms. Factors associated with rehospitalization and treatment adherence and variables predicting rehospitalization and treatment adherence were investigated.

The study was approved by the Ethics Committee of Giresun Training and Research Hospital (Approval number: 09.04.2025/09)

### 2.2 Statistical analyses

Statistical analyses were conducted using IBM SPSS Statistics 27. The normality of the data distribution was assessed using the Shapiro-Wilk test. Categorical variables were presented as frequency (n) and percentage (%), normally distributed numerical variables as mean and standard deviation (SD), and non-normally distributed numerical variables as median and interquartile range (IQR: first quartile–third quartile). The Chi-square test, Student’s t-test, or Mann-Whitney U test was used for pairwise group comparisons, depending on the data type. Binary logistic regression analyses were conducted to identify the factors predicting rehospitalization and treatment adherence, and odds ratios (ORs) were reported. Additionally, since patients were not followed for the same duration, Cox regression analyses were performed, accounting for follow-up times and variables that could be influenced by follow-up duration. Hazard ratios (HRs) were reported. A p-value < 0.05 was considered statistically significant.

## 3 Results

Between January 2022 and February 2025, a total of 106 patients were found to have undergone regular psychiatric evaluations as part of mandatory forensic psychiatric follow-up in the psychiatric outpatient clinic. Of these, 38 patients were excluded because they had a diagnosis other than a psychotic disorder. Our study sample consisted of 68 individuals diagnosed with psychotic disorders. In the sample, the mean age at the initiation of mandatory forensic psychiatric evaluations was 46.76 years (SD: 10.23), the mean PDC was 0.85 (SD: 0.16), and the mean follow-up duration was 50.49 months (SD: 39.23). Among the patients, 55 (80.9%) were male, 28 (41.2%) received both oral and LAI antipsychotic treatment, 17 (25%) were under regular CMHC follow-up, 30 (44.1%) attended follow-ups every three months, 38 (55.9%) attended follow-ups every six months, and 48 (70.6%) were single. A total of 48 patients (70.6%) were adherent to treatment (PDC ≥ 0.8). During the follow-up period, 10 patients (14.7%) were rehospitalized due to violent behavior, while 58 patients (85.3%) remained hospitalization-free until the study endpoint. Of the 58 patients, 54 are still under follow-up. The mandatory forensic psychiatric follow-up was discontinued for four patients due to significant symptom improvement, and they were transitioned to standard outpatient psychiatric care. All four patients who showed improvement were adherent to treatment, with a mean PDC of 0.92 (SD: 0.05), and three of them were receiving LAI antipsychotic treatment.

The comparison of patients who were rehospitalized due to violent behavior and those who did not exhibit violent behavior and, therefore, were not rehospitalized in terms of age, gender, marital status, treatment type, regular CMHC follow-up, follow-up frequency, and treatment adherence, is presented in **Table 1**. No statistically significant differences were found in age, gender, or marital status. Patients receiving LAI antipsychotic treatment, those under regular CMHC follow-up, and those with three-month follow-up intervals had lower rehospitalization rates; however, these differences were not statistically significant (p > 0.05). When treatment adherence was evaluated, the adherent group had a significantly lower rehospitalization rate than the non-adherent group (p < 0.001). The PDC value was significantly higher in the non-rehospitalized group (p < 0.001).

**Table 1:** Comparison of demographic and clinical characteristics between rehospitalized and non-rehospitalized patients.

Demographic and clinical variables	Mean ± standard deviation	Number of subjects (%)	Median (First–Third Quartile)	P
Age	47.07 ± 10.02	45.00 ± 11.80	t=0.588	0.559
Gender			X²=0.006	0.939
Female	11 (19%)	2 (20%)		
Male	47 (81%)	8 (80%)		
Treatment			X²=2.171	0.179
Oral	32 (55.2%)	8 (80%)		
LAI	26 (44.8%)	2 (20%)		
CMHC follow-up			X²=0.156	0.693
No	43 (74.1%)	8 (80%)		
Yes	15 (29.1%)	2 (20%)		
Follow-up frequency			X²=0.081	0.776
Three-month	26 (44.8%)	4 (40%)		
Six-month	32 (55.2%)	6 (60%)		
Marital status			X²=0.002	0.965
Single	41 (70.7%)	7 (70%)		
Married	17 (29.3%)	3 (30%)		
Treatment adherence			X²=14.452	<0.001
Non-adherent	12 (20.7%)	8 (80%)		
Adherent	46 (79.3%)	2 (20%)		
PDC	0.92 (0.82-0.97)	0.62 (0.44-0.78)	U=101.500	<0.001

t, Independent samples t-test statistic; U, Mann–Whitney U test statistic; X², Chi-square test statistic; p < 0.05 was considered statistically significant.

LAI, Long-acting injectable; CMHC, Community mental health centers; PDC, Proportion of days covered.

Bold values indicate statistically significant results (p < 0.05).

The results of the binary logistic regression analyses conducted to identify variables predicting rehospitalization due to violent behavior are presented in **Table 2**. The table reports univariate ORs with 95% confidence intervals (CIs). The results indicate that age, gender, and marital status had no significant predictive effect. Although LAI treatment, regular CMHC follow-up, and three-month follow-up intervals were associated with a reduced risk of rehospitalization, these effects were not statistically significant (p > 0.05PDC was found to be significantly and inversely associated with rehospitalization (B = -11.762, p < 0.001, OR < 0.001). The results indicate that the rehospitalization rate decreases significantly as PDC value increases. Similarly, lack of treatment adherence increased the risk of rehospitalization by approximately 15 times (p < 0.001, OR = 0.065).

**Table 2:** Binary logistic regression analysis predicting rehospitalization due to violent behavior.

Predictive variables	Reference	B	SE	Wald	P	OR	95% CI	R²
PDC	–	**-11.762**	**3.344**	**12.370**	**<0.001**	**<0.001**	**<0.001-0.005**	**0.530**
Age	–	-0.020	0.034	0.352	0.553	0.980	0.917-1.047	0.009
Treatment adherence	Non-adherent	**-2.730**	**0.854**	**10.209**	**0.001**	**0.065**	**0.012-0.348**	**0.313**
Gender	Female	-0.066	0.859	0.006	0.939	0.936	0.174-5.037	0.000
Treatment	Oral	-1.179	0.833	2.000	0.157	0.308	0.060-1.576	0.060
CMHC follow-up	No	-0.333	0.846	0.155	0.694	0.717	0.137-3.759	0.004
Follow-up frequency	Three-month	0.198	0.697	0.080	0.777	1.219	0.311-4.781	0.002
Marital status	Single	0.033	0.748	0.002	0.965	1.034	0.239-4.477	0.000

B, Unstandardized regression coefficient; SE, Standard error; Wald, Wald Chi-square test statistic; OR, Odds ratio; CI, Confidence interval; R², Nagelkerke R-squared; p < 0.05 was considered statistically significant.

PDC, Proportion of days; CMHC, Community mental health centers.

Bold values indicate statistically significant results (p < 0.05).

There were no statistically significant differences between adherent and non-adherent patients in terms of age (p = 0.583), gender (p = 0.905), marital status (p = 0.514), or follow-up frequency (p = 0.925). Higher medication adherence rates were found in patients receiving LAI antipsychotic treatment (p = 0.227) and those under regular CMHC follow-up (p = 0.219), but these results were not statistically significant.

According to the results of the regression analysis conducted to determine the effects of variables that could predict treatment adherence, age (p = 0.578), gender (p = 0.905), marital status (p = 0.515), and follow-up frequency (p = 0.925) had no significant predictive effect. Although LAI antipsychotic treatment (p = 0.231) and regular CMHC follow-up (p = 0.228) were associated with increased medication adherence compared to oral treatment and the absence of regular CMHC follow-up, these effects were not statistically significant.

Since patients were not followed simultaneously or for the same duration, Cox regression analyses were performed, accounting for follow-up durations. In the Cox regression analysis, where the dependent variable was rehospitalization due to violent behavior, it was found that LAI antipsychotic treatment (HR = 0.347, 95% CI = 0.073-1.637), regular CMHC follow-up (HR = 0.740, 95% CI = 0.157-3.495), and three-month follow-up intervals (HR = 1.452, 95% CI = 0.406-5.198) were associated with a reduced risk of rehospitalization, but these effects were not statistically significant.

## 4 Discussion

This study investigated the association between post-discharge treatment adherence, regular psychiatric follow-up, and prescribed treatments with the risk of rehospitalization due to recurrent violent behavior among individuals previously involuntarily hospitalized for violence. In patients with psychotic disorders under mandatory forensic psychiatric follow-up, in-depth research on the role of treatment adherence in predicting violence-related rehospitalization remains limited. By statistically controlling for various clinical and demographic factors, this study highlights the significance of treatment adherence in this unique population and provides novel contributions to the existing literature.

Approximately 71% of all patients in the study sample were adherent to treatment. This rate is higher than the overall treatment adherence rates previously reported in patients with psychotic disorders and post-discharge treatment adherence rates (15–17). In our sample, psychiatric evaluations and treatments are not left to patients’ initiative. They are mandatorily followed under the framework of legal regulations. The forensic nature of the follow-up process may motivate patients to adhere to their treatment appropriately. The forensic aspect of the follow-up process and the fact that patients are regularly monitored may explain the high treatment adherence rates. The relationship between involuntary hospitalization and treatment adherence is complex (18). Patients may feel pressured during treatment following involuntary hospitalization (19). De Haan et al. (20) provided evidence suggesting that involuntary hospitalization may reduce treatment adherence in the long term. However, in their study, unlike ours, they evaluated first-episode schizophrenia patients. In first-episode psychosis, treatment non-adherence can be observed at high levels (21). In our study, the focus was primarily on chronic psychotic disorder patients. In contrast, Kortrijk et al. (22) found that involuntary hospitalization was associated with increased patient treatment motivation.

In the retrospective follow-up of our study, it was found that approximately 15% of patients were rehospitalized due to violent behavior. In a historical cohort study conducted with 6520 patients diagnosed with various psychiatric disorders, it was found that 69% of patients discharged from forensic psychiatry services were rehospitalized, and 40% reoffended (23). Their study’s average follow-up duration of 15.6 years is considerably longer than our study’s 4.2 years follow-up period. The results highlight the increased risk of recurrent violent behavior over time in patients with a history of hospitalization in forensic psychiatry services. In another study, it was reported that approximately half of forensic psychiatric patients were rehospitalized during a 2–7 year follow-up period (24). However, these hospitalizations were not solely due to violent behavior but occurred for various reasons. Another study reported a rehospitalization rate of 28% within 12 months, with substance use being an important determinant (25). In a study with an average follow-up duration of 12 months, it was found that 6% of patients who were under forensic follow-up after discharge reoffended (26). The findings suggest that the time elapsed after discharge is critical in terms of the risk of recurrent violent behavior and that careful assessment and interventions are required at every stage.

In our study, no significant differences were found in violence-related rehospitalization rates across diagnostic groups, and no direct association was observed between diagnosis and the recurrence of violent behavior. This finding is consistent with the existing literature. In a comprehensive systematic review by Whiting et al. (27), prior aggression and substance use, rather than diagnostic category, were identified as the strongest predictors of violence over five- to ten-year follow-up periods in individuals with schizophrenia spectrum disorders. Moreover, violent behavior is often closely associated with the severity of psychotic symptoms (28). Nonetheless, the unique protective effect of clozapine in reducing violent recidivism has been repeatedly emphasized in the literature. Faden and Citrome (1) described clozapine as the gold standard in the treatment of persistent aggression in treatment-resistant schizophrenia and highlighted that its anti-aggressive effects may be independent of its antipsychotic properties. However, in our study, the small sample size and the very limited number of patients using clozapine may have precluded the detection of diagnostic differences or the specific impact of clozapine in the statistical analyses.

There is significant evidence indicating that LAI antipsychotic use contributes to treatment adherence in patients with psychotic disorders and reduces hospitalization rates (29, 30). Additionally, LAI antipsychotics have been associated with a reduction in violent behavior (31). However, data on LAI antipsychotic use in the forensic population are limited (32). It has previously been reported that CMHC services reduce rehospitalization rates (33). However, there is no direct research on the impact of CMHC services on violent behavior. Furthermore, while regular and frequent follow-ups have been shown to improve symptoms and reduce rehospitalization rates (34), no study has been found that evaluates the effect of follow-up frequency in patients under forensic follow-up. In our study, patients receiving LAI antipsychotic treatment had lower rehospitalization rates than those receiving oral treatment, and patients with regular CMHC follow-up had lower rehospitalization rates than those without it. Additionally, patients who followed every three months had lower rehospitalization rates than those who followed every six months. These variables were found to reduce the risk of rehospitalization. Since the duration of follow-up may also affect the outcome variable, the Cox regression analysis similarly found that LAI antipsychotic use, regular CMHC follow-up, and more frequent follow-up reduced the risk of rehospitalization. However, these effects were not statistically significant. Due to the small sample size, these results may reflect a situation where statistical power is low. Therefore, the accuracy and validity of these findings should be reassessed through studies conducted with larger sample sizes.

LAI and oral antipsychotics are generally considered to share similar neurobiological mechanisms of action, primarily dopamine D_2_ receptor blockade and, for second-generation antipsychotics, additional 5-HT_2_A antagonism. However, their primary difference lies in their pharmacokinetic profiles (35, 36). LAI formulations provide predictable and stable plasma drug concentrations through slow release and depot effects (37). In contrast, oral antipsychotics administered daily result in peak–trough fluctuations, leading to intermittent and unstable plasma levels and D_2_ receptor occupancy. By promoting pharmacokinetic stability, LAI antipsychotics enable more continuous and balanced neurochemical effects on dopaminergic and serotonergic systems (38). For this reason, LAI treatment is considered a valuable option, particularly for patients with poor treatment adherence, frequent relapses, or a history of violent behavior. Furthermore, LAI antipsychotics have been shown to be effective in early psychosis in terms of improving adherence, reducing relapse risk, and enhancing symptom outcomes, with efficacy comparable to oral treatments (39, 40). Although our study did not include patients in the early phase of psychosis, the elevated risk of violence in this population (41) highlights the need for further research within forensic psychiatry contexts.

One of the main findings of our study is that patients with treatment adherence had fewer rehospitalizations than those with treatment non-adherence. In connection with this, the PDC value was found to be significantly higher in the non-rehospitalized group due to violent behavior. In patients with psychotic disorders, the improvement of symptoms and clinical features related to the disorder is largely dependent on treatment adherence (42). There is a significant relationship between treatment adherence and rehospitalization in patients with psychotic disorders. This relationship is critical at the beginning of treatment and throughout the entire follow-up period (43). In a study conducted with schizophrenia patients, similar to the results of our study, it was found that individuals with higher PDC values had lower hospitalization rates (44). Similarly, in forensic psychiatry patients, treatment non-adherence is an important variable determining rehospitalization rates (25). Psychosocial interventions aimed at improving treatment adherence show promise in the treatment of psychotic disorders, and considering their impact on symptoms and rehospitalization, they appear to be worth exploring (45). Our study, consistent with the literature, found that the PDC value strongly predicted the rehospitalization risk. Lack of treatment adherence increased the rehospitalization risk by approximately 15 times. These results underscore the importance of treatment adherence in forensic psychiatric patients.

Considering the impact of treatment adherence on reducing both symptoms and rehospitalizations in patients with psychotic disorders, evaluating the factors that influence treatment adherence in the forensic population also appears to be crucial. No predictive effect of follow-up frequency on treatment adherence was identified. No study has directly investigated the relationship between follow-up frequency and medication adherence. This area requires further research. We found a higher rate of medication adherence in patients receiving LAI antipsychotic treatment and those under regular CMHC follow-up. Additionally, it was found that LAI antipsychotic use and being under regular CMHC follow-up increased medication adherence rates. However, these effects were not statistically significant. LAI antipsychotic medications may contribute to improving treatment adherence in patients with psychotic disorders by eliminating the need for daily dosing, preventing missed doses, and causing fewer side effects (46). A recent study conducted in Turkey reported that LAI treatment in forensic psychotic disorder patients is associated with better improvement in medication adherence compared to oral treatment (47). CMHC services are crucial in increasing medication adherence in patients (48). Studies on this topic have been conducted in non-forensic psychiatry patients. The lack of statistical significance for these effects in our study is likely due to the small sample size.

Although the number of patients is insufficient for statistical analysis, the characteristics of the four patients whose mandatory forensic evaluations were discontinued due to significant symptom improvement and reduced danger to others are noteworthy. All four patients were adherent to treatment, and three of them were using LAI antipsychotic medications. The characteristics of the patients are consistent with the literature regarding treatment adherence and LAI antipsychotics.

Beyond its forensic implications, the findings of this study underscore the clinical relevance of treatment-related factors in the management of psychotic disorders. Independent of legal status, non-adherence to antipsychotic treatment is a well-established predictor of relapse and rehospitalization in patients with schizophrenia and related disorders (43). Clinical interventions such as the use of LAI antipsychotics not only improve treatment adherence but have also been associated with reductions in aggression and symptom severity (31). Similarly, structured community-based psychiatric services play a critical clinical role by facilitating continuity of care, promoting functional recovery, and supporting treatment engagement (12). These approaches are essential not only in forensic settings but also in routine psychiatric care. Thus, the current findings may inform broader clinical strategies aimed at reducing violent behavior and improving outcomes in individuals with chronic psychotic disorders.

While most existing research focuses on psychotic patients in the general population, our study is one of the few to thoroughly examine the relationship between treatment adherence and violent behavior in the forensic patient population. In addition, our study has some limitations. We did not conduct face-to-face evaluations of the patients, and the data were collected retrospectively. For this reason, we were unable to examine certain variables that could be related to rehospitalization, such as substance use. For instance, factors like cognitive function, psychopathological trajectories, insight, education level, and sociocultural characteristics were not addressed. Although we explored marital status, we could not fully assess social support. These factors may influence treatment adherence and the risk of recurrent violent behavior and, therefore, can be considered uncontrolled potential confounders in the study. Although calculating PDC is an accepted method in the literature for measuring medication adherence, we do not know whether the patients took their medications, as we did not conduct blood tests to verify this. Additionally, our knowledge is limited regarding whether the patients engaged in violent behavior outside the awareness of the clinician, local law enforcement, or family members. Finally, the study was conducted at a single center with a small sample size, which may limit the generalizability of the results. Due to these limitations, the findings should be interpreted with caution.

## 5 Conclusion

Investigating the rehospitalization risk in patients with psychotic disorders and a history of violent behavior is crucial for improving both individual patient care and public safety. This study aims to provide valuable insights into this critical issue for clinicians and policymakers.

Our findings highlight the critical role of treatment adherence in preventing recurrent hospitalizations. A 15-fold increase in the rehospitalization risk was observed in non-adherent patients. Additionally, while LAI antipsychotic use, regular follow-up at CMHCs, and more frequent psychiatric monitoring appear to be associated with lower rehospitalization rates, these effects likely did not reach statistical significance due to the small sample size. This study emphasizes the importance of structured psychiatric follow-up programs in forensic psychiatric populations, particularly in ensuring medication adherence. Given that treatment adherence is a key modifiable factor, interventions targeting adherence, such as the use of LAI antipsychotics and psychosocial interventions, should be prioritized to reduce the risk of rehospitalization related to violence.

In future research, rehospitalization and other clinical outcomes could be considered for evaluation. For example, outcomes such as recurrence of violent incidents, reoffending rates, suicide attempts, or changes in overall quality of life and social functioning could be monitored. This would allow for a better understanding of the impact of post-involuntary hospitalization follow-up programs on all aspects of patients’ lives. Future studies should include objective measures of medication adherence, address potential confounding factors, and use larger multicenter cohorts to enhance the generalizability of the findings. Additionally, more extended follow-up periods should be planned to investigate the long-term effects that may emerge over time. Examining which factors sustainably impact over the long term will contribute to developing improved care models.

## Data Availability

The raw data supporting the conclusions of this article will be made available by the authors, without undue reservation.
